# A proof-of-concept study to construct Bayesian network decision models for supporting the categorization of sudden unexpected infant death

**DOI:** 10.1038/s41598-022-14044-w

**Published:** 2022-06-13

**Authors:** Hideki Hamayasu, Masashi Miyao, Chihiro Kawai, Toshio Osamura, Akira Yamamoto, Hirozo Minami, Hitoshi Abiru, Keiji Tamaki, Hirokazu Kotani

**Affiliations:** 1 Department of Forensic Medicine, Kyoto University Graduate School of Medicine, Kyoto, Japan; 2 Department of Pediatrics, Japanese Red Cross Kyoto Daini Hospital, Kyoto, Japan; 3 Center for Medical Education, Kyoto University Graduate School of Medicine, Kyoto, Japan; 4 Department of Forensic Medicine and Sciences, Mie University Graduate School of Medicine, 2-174 Edobashi, Tsu, Mie 5148507 Japan

**Keywords:** Epidemiology, Paediatric research, Medical research, Risk factors

## Abstract

Sudden infant death syndrome (SIDS) remains a leading cause of infant death in high-income countries. Supporting models for categorization of sudden unexpected infant death into SIDS/non-SIDS could reduce mortality. Therefore, we aimed to develop such a tool utilizing forensic data, but the reduced number of SIDS cases renders this task inherently difficult. To overcome this, we constructed Bayesian network models according to diagnoses performed by expert pathologists and created conditional probability tables in a proof-of-concept study. In the diagnostic support model, the data of 64 sudden unexpected infant death cases was employed as the training dataset, and 16 known-risk factors, including age at death and co-sleeping, were added. In the validation study, which included 8 new cases, the models reproduced experts’ diagnoses in 4 or 5 of the 6 SIDS cases. Next, to confirm the effectiveness of this approach for onset prediction, the data from 41 SIDS cases was employed. The model predicted that the risk of SIDS in 0- to 2-month-old infants exposed to passive smoking and co-sleeping is eightfold higher than that in the general infant population, which is comparable with previously published findings. The Bayesian approach could be a promising tool for constructing SIDS prevention models.

## Introduction

Sudden infant death syndrome (SIDS) remains a leading cause of infant death in high-income countries^1,2^. SIDS, as described in the San Diego definition^3^, refers to the sudden unexpected death of an infant aged less than 1 year. The onset of the fatal episode tends to occur during sleep and is attributed to SIDS if the cause remains unexplained after a thorough investigation, including a complete autopsy, review of the circumstances of death, and clinical history. SIDS-associated mortality peaked in the 1980s and decreased in the 1990s. This has been predominantly attributed to the “Back to Sleep” campaigns that promoted the supine sleeping position^1^. However, the declining rate of SIDS-associated mortality has plateaued in recent decades^1,2^. Continuous and evolving research may contribute to further reduction in SIDS.

A major factor contributing to the persistence of SIDS is the difficulty in diagnosing SIDS^4,5^. To establish effective preventive measures for a disease, the diagnosis must be unified both internationally and regionally. However, there are concerns that the diagnosis of SIDS is inconsistent across nations and professions^4,6,7^. In addition, cases that would have previously been reported as SIDS are currently reported as “undetermined” or “asphyxia,” although the overall number of infants dying suddenly and unexpectedly during sleep has remained constant^8–10^. This diagnostic shift has resulted in a reduction in SIDS incidence and a concomitant increase in the incidence of “undetermined” and “asphyxia” cases. The diagnostic shift and inconsistencies in diagnosis have impeded effective comparisons across epidemiological studies and have hindered the development of effective preventive measures^11^. The Centers for Disease Control and Prevention and the American Academy of Pediatrics defined sudden unexpected infant death (SUID) as a term that combines three categories of infant death in the International Classification of Diseases, 10th Revision (ICD-10), i.e., SIDS (R95), ill-defined or unknown causes (R99), and accidental suffocation and strangulation in bed (W75); this term is recommended for use in epidemiological studies^12,13^. However, this is an umbrella term for unexpected deaths, including unexplained (R95 & R99) and explained deaths (W75). Therefore, it is confusing from a diagnostic perspective. Nevertheless, developing preventive measures for SIDS requires a diagnostic method that enables the distinction of SIDS from SUID.

Another factor contributing to the persistence of SIDS is the difficulty in predicting the onset of SIDS early after birth. Indeed, effective SIDS prediction measures have yet to be developed^14,15^. The risk of SIDS differs among individual children, and interactions between risk factors may modulate the risk of developing SIDS. Big data and artificial intelligence technology may facilitate the elucidation of complex relationships between risk factors and the development of accurate onset predictive and diagnostic models^15,16^. However, the number of SIDS cases per institution is generally low, and even if cases are compiled from multiple institutions, the quality of data is low owing to the aforementioned inconsistencies and shift in diagnosis. To resolve these issues, we hypothesized that a Bayesian approach would provide a powerful tool for creating SIDS diagnostic or onset-predictive support models because of its strengths and flexibility with small sample size studies^17^.

Therefore, we aimed to predict the cause of death and onset of SUID through a Bayesian diagnostic and onset-predictive support model for SIDS. In this proof-of-concept study, we first examined the current state of SUID diagnosis in Japan using statistics from the Ministry of Health, Labour and Welfare^18^, which includes the cause-of-death classification of sudden infant death by prefecture, to clarify whether the cause-of-death statistics in Japan could be used to construct SIDS prediction models. We then constructed Bayesian models through experts’ construction of networks with software-assisted conditional probability table creation to investigate whether diagnostic and onset-predictive support models could be constructed using Bayesian inference based on data, even with a small number of cases, from forensic autopsy cases with expert consensus diagnosis and detailed information.

## Results

### Variance in SIDS diagnosis among different regions in Japan

**Figure 1:**
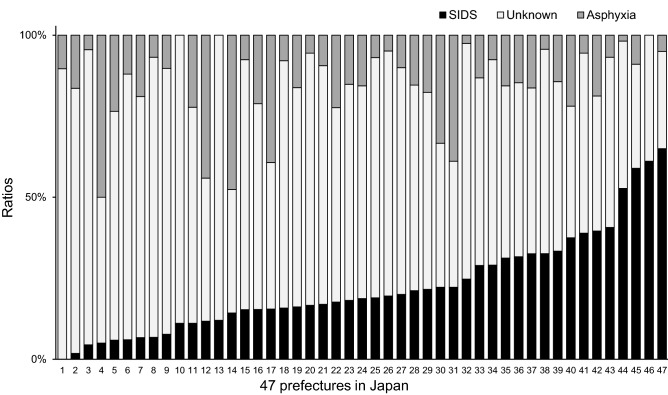
Ratios of sudden unexpected infant death (SUID) subcategories to total SUID cases in the 47 prefectures in Japan from 2012 to 2018. SUID was defined as a set of the following three subcategories: SIDS, accidental asphyxia, and unknown causes of death. SIDS, R95 (SIDS) in ICD-10; accidental asphyxia, the combination of W75 (accidental suffocation and strangulation in bed), W78 (inhalation of gastric contents), and W79 (inhalation and ingestion of food, causing obstruction of respiratory tract); unknown causes of death, the combination of R96 (other sudden death, cause unknown), R98 (unattended death), and R99 (other ill-defined and unspecified causes of mortality). SUID, sudden unexpected infant death; ICD-10, International Classification of Diseases, 10th Revision; SIDS, sudden infant death syndrome.

To investigate potential interregional variance in SIDS diagnosis according to the SUID classification in Japan, we first compared the subcategories of SUID cases among 47 prefectures. From 2012 to 2018 in Japan, there were 6,917,706 live births, 13,917 total infant deaths (2.0/1000 live births), and SUID accounted for 25% of infant deaths. The SIDS ratio was highly variable among different regions (Fig. 1). Among 47 regions, only seven had an SIDS incidence greater than 0.2/1000 live births (Supplementary Fig. S1), which is the lowest incidence rate of SIDS recorded in high-income countries^4,9^. In total, 26 regions had an SIDS incidence of less than 0.1/1000 live births, and most of them had a higher ratio of unknown causes of death or accidental asphyxia. As shown in Table 1, the coefficient variation value (an indicator of the extent of variation) of total SUID cases was 18.4%, which was considerably lower than that of SIDS (72.7%).

**Table 1:** The variability in the diagnosis of the three SUID diagnostic subcategories.

	Mean	SD	CV (%)
SIDS	0.11	0.08	72.7
Asphyxia	0.07	0.05	71.4
Unknown	0.31	0.10	32.2
SUID	0.49	0.09	18.4

The infant mortality rate per 1000 live births was calculated for 47 prefectures in Japan between 2012 and 2018. The CV was calculated by the SD by the mean. CV, coefficient of variation; SD, standard deviations; SIDS, sudden infant death syndrome; SUID, sudden unexpected infant death.

Collectively, the proportion of diagnostic subcategories consisting of SIDS, unknown causes of death, and accidental asphyxia was uneven, although the total proportion of SUID cases in Japan did not differ substantially among the 47 prefectures.

### Analysis of autopsy cases for model construction

**Figure 2:**
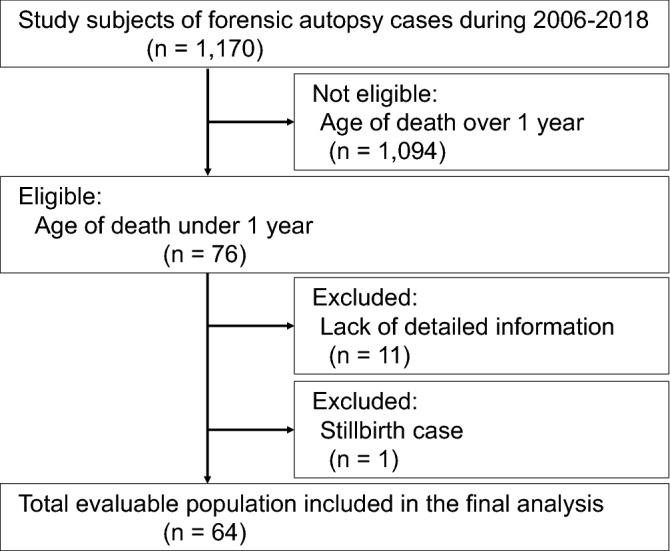
Flow chart of case enrollment and exclusion.

In total, 1170 autopsy cases were reported in Kyoto University between January 2006 and December 2018 (Fig. 2). Overall, 1094 cases were ineligible because of death after 1 year of age. Among the remaining 76 eligible cases, 11 were excluded because of the lack of detailed information, and 1 was excluded due to stillbirth. A total of 64 SUID cases were included in our analysis.

The proportion of SIDS cases accounted for 64% of the total cases (41 of 64 cases, Table 2). Other causes of death included various internal and external disorders such as peritonitis (3.1% of cases), accidental asphyxia, and congenital anomalies (7.8% of cases).

**Table 2:** Causes of death in 64 infant autopsy cases.

	Total (n = 64)	Male (n = 36)	Female (n = 28)
	n (%)	n (%)	n (%)
**Internal causes**	52 (81.2)	27 (75.0)	25 (89.3)
SIDS	41 (64.0)	22 (61.0)	19 (67.9)
Pneumonia	4 (6.3)	2 (5.6)	2 (7.1)
Peritonitis	2 (3.1)	1 (2.8)	1 (3.6)
Congenital anomaly^a^	5 (7.8)	2 (5.6)	3 (10.7)
**External causes**	12 (18.8)	9 (25.0)	3 (10.7)
Abuse (AHT)	4 (6.3)	3 (8.3)	1 (3.6)
Asphyxia	5 (7.8)	3 (8.3)	2 (7.1)
Drowning	3 (4.7)	3 (8.3)	0 (0.0)

^a^Congenital anomaly includes an anomalous origin of the coronary artery, a complex congenital heart disease, and an alveolar capillary dysplasia. AHT, abusive head trauma; SIDS, sudden infant death syndrome.

Table 3 presents the demographic characteristics of the 64 cases, including known risk factors for SIDS^19–21^. Of the 16 risk factors, only sleeping-related death and co-sleeping showed statistically significant differences for SIDS incidence.

**Table 3:** Demographic characteristics in 64 infant autopsy cases.

	Total (n = 64)		SIDS (n = 41)		Others (n = 23)		*P* value
	Mean (SD)	n (%)	Mean (SD)	n (%)	mean (SD)	n (%)	
**Age of death, months**	4.5 (2.8)		4.4 (2.5)		4.6 (3.2)		
0–2 months		22 (34.4)		13 (31.7)		9 (39.1)	0.11^a^
3–6 months		32 (50.0)		24 (58.5)		8 (34.8)	
7–11 months		10 (15.6)		4 (9.8)		6 (26.1)	
**Sex**							
Male		36 (56.2)		22 (53.7)		14 (60.8)	0.61^b^
Female		28 (43.8)		19(46.3)		9 (39.2)	
**Gestational age, weeks**	38.5 (2.2)		38.1(2.5)		39.0 (1.5)		
< 37 weeks		5 (7.8)		4 (9.8)		1 (4.4)	0.65^b^
≥ 37 weeks		59 (92.2)		37 (90.2)		22 (95.6)	
**Birth weight, g**	2843 (491)		2820 (534)		2882 (414)		
< 2500 g		13 (20.3)		10 (24.4)		3 (13.0)	0.35^b^
≥ 2500 g		51 (79.7)		31 (75.6)		20 (87.0)	
**Sleep related death**							
Yes		54 (84.4)		41 (100.0)		13 (56.5)	< 0.001^b^
With co-sleeping		37 (57.8)		32 (78.0)		5 (21.7)	0.01^b^
Without co-sleeping		17 (26.6)		9 (22.0)		8 (34.8)	
Sleeping position							
Supine		20 (31.2)		17 (41.5)		3 (13.0)	0.06^a^
Prone		23 (36.0)		19 (46.3)		4 (17.5)	
Other position		5 (7.8)		2 (4.9)		3 (13.0)	
Unknown position		6 (9.4)		3 (7.3)		3 (13.0)	
No		10 (15.6)		0 (0.0)		10 (43.5)	
**Maternal age, years**	30.0 (7.0)		29.9 (7.2)		30.3 (6.7)		
< 19		5 (7.8)		4 (9.8)		1 (4.4)	0.85^a^
20–34		38 (49.4)		24 (58.5)		14 (60.8)	
≥ 35		21 (32.8)		13 (31.7)		8 (34.8)	
**Number of siblings**	1.3 (1.3)		1.4 (1.5)		0.9 (0.8)		
0		19 (29.7)		11 (26.8)		8 (34.8)	0.44^a^
1		25 (39.1)		15 (36.6)		10 (43.5)	
≥ 2		20 (31.2)		15 (36.6)		5 (21.7)	
**History of siblings with SUID**							
Yes		2 (3.1)		2 (4.9)		0 (0.0)	0.53^b^
No		62 (96.9)		39 (95.1)		23 (100.0)	
**Breastfeeding**							
Yes		32 (50.0)		21 (51.2)		11 (47.8)	1^b^
No		30 (46.9)		20 (48.8)		10 (43.5)	
Unknown		2 (3.1)		0 (0.0)		2 (8.7)	
**Passive smoking**							
Yes		30 (46.9)		21 (51.2)		9 (39.1)	1^b^
No		12 (18.7)		9 (22.0)		3 (13.0)	
Unknown		22 (34.4)		11 (26.8)		11 (47.9)	
**Alcohol influence**^**c**^							
Yes		9 (14.1)		4 (9.8)		5 (21.7)	0.26^b^
No		55 (85.9)		37 (90.2)		18 (78.3)	
**Vaccination**^**d**^							
Yes		19 (29.7)		10 (24.4)		9 (39.1)	0.25^b^
No		43 (67.2)		30 (73.2)		13 (56.5)	
Unknown		2 (3.1)		1 (2.4)		1 (4.4)	
**Maltreatment**							
Yes		25 (39.0)		19 (46.3)		6 (26.1)	0.18^b^
No		39 (61.0)		22(53.7)		17 (73.9)	
**Infectious disease findings**							
Clinical symptoms							
Yes		11 (17.2)		10 (24.4)		1 (4.4)	0.08^b^
No		53 (82.8)		31 (75.6)		22 (95.6)	

^a^Fisher–Freeman–Halton exact test.

^b^Fisher’s exact test.

^c^Alcohol influence during childcare with caregivers.

^d^Vaccination within 1 month before death. SIDS, sudden infant death syndrome.

Complete information about the 64 cases, including the clinical history and infant sleeping environment, was obtained from the police. For example, the information about infant sleep environment included photographs of baby beds, blankets, and pillows. The properties and thickness of the mattresses were described, along with a photo that indicated the dimensions using a scale. Moreover, there were photographs and illustrations of reconstruction of the death scene with the infants’ caregivers in almost all cases.

There was some missing information for 5 of the 16 risk factors, indicated as “unknown” in Table 3: sleep position, 6 of 64 cases; breastfeeding, 2 cases; vaccination, 2 cases; and passive smoking, 22 cases. The main cause of missing information about passive smoking was lack of obtaining information at the beginning of the research.

Histological examinations were performed in all cases, as described in the “Methods” section. Among the 41 cases of SIDS, 2 showed mild lymphocytic infiltrate in the alveoli; however, the amount was not indicative of pneumonia. Meanwhile, 4 cases of pneumonia and 2 of peritonitis showed significant inflammatory findings on histology in the organs involved. Among the 5 congenital anomalies, except for pulmonary capillary dysplasia, 4 were confirmed by gross autopsy findings; a case of pulmonary capillary dysplasia was confirmed by histological findings. No significant inflammatory findings were found in the 12 cases in which death occurred due to external causes, except for a mild lymphocytic infiltration in the alveoli in one case.

Rapid antigen test for infections was performed in 53 of 64 cases; 9 of 53 cases (6 cases of death due to SIDS, 3 due to other internal causes, and none due to external causes) were positive for respiratory syncytial virus but negative for all other pathogens.

### SIDS diagnostic support models to reduce the risk of diagnostic bias

**Figure 3:**
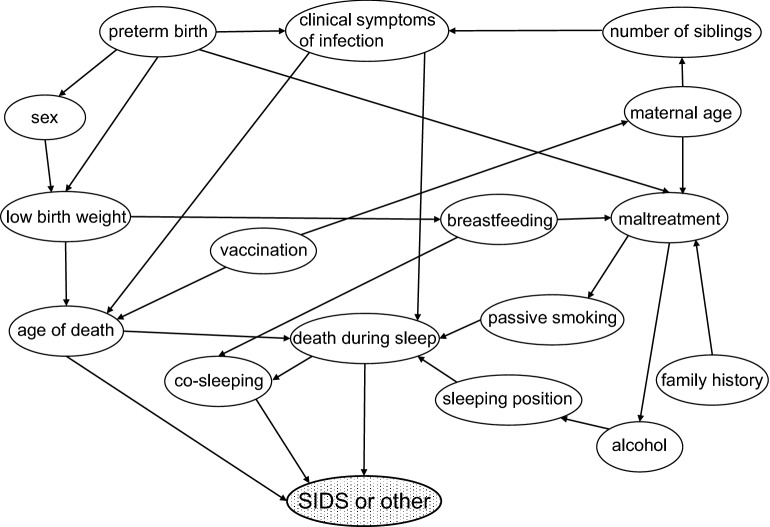
A Bayesian diagnostic support model for SIDS. This model reflects the relationship among risk factors leading to death (cf. Supplementary Fig. S2). A conditional probability table was created for each factor. Including the presence or absence of each factor enables the calculation of SIDS diagnosis probability. SIDS, sudden infant death syndrome.

To examine the utility of Bayesian approach for developing SIDS diagnostic support models, we applied 16 risk factors to Bayesian networks, regardless of statistically significant differences, and constructed three types of diagnostic support models as described in the Methods section (Fig. 3: Model 1; Supplementary Fig. S2: Models 2 and 3). In all models, known high-risk factors such as “age of death,” “co-sleeping,” and “death during sleep” were consequently located close to a SIDS node. In contrast, “breastfeeding,” “preterm birth,” and “male sex,” which are also known high-risk factors, were located far from a SIDS node in these models.

A validation cohort comprising six SIDS cases, two cases with other internal causes, and no cases with external causes was used to validate the usefulness and limitations of the models. Table 4 presents the SIDS or other diagnostic probabilities estimated using the three models, consensus diagnoses made by experts, and demographic characteristics of each case. In the validation study, all three models identified SIDS as the most likely diagnosis for four or five of the six SIDS cases (cases 2, 3, 5, 6, and 8). However, the results obtained for cases 1, 4, and 7 did not match the expert diagnoses. The probabilities provided by Model 3 were closer to the consensus diagnoses of experts than those provided by the other two models. Notably, the probability values in these cases ranged from 0.71 to 0.87, indicating the uncertainty of SIDS diagnosis by the experts.

**Table 4:** Probabilities estimated by diagnostic support models in eight validation cases.

Validation cohort	Case-1	Case-2	Case-3	Case-4	Case-5	Case-6	Case-7	Case-8
Diagnosis by experts	Internal	SIDS	SIDS	Internal	SIDS	SIDS	SIDS	SIDS
Model 1								
(SIDS)	0.70	0.70^a^	0.70^a^	0.92	0.40	0.89^a^	0.33	0.92^a^
Model 2								
(SIDS)	0.64	0.69^a^	0.69^a^	0.88	0.50	0.91^a^	0.22	0.85^a^
Model 3								
(SIDS)	0.64	0.71^a^	0.71^a^	0.84	0.74^a^	0.87^a^	0.34	0.87^a^
(Internal)	0.26	0.11	0.11	0.01	0.12	0.006	0.48	0.03
(External)	0.10	0.18	0.18	0.15	0.13	0.12	0.17	0.10
Age of death, months	6	6	5	1	10	6	8	0
Sex	F	F	M	F	M	F	F	F
Gestational age, weeks	≥ 37	≥ 37	≥ 37	≥ 37	≥ 37	≥ 37	≥ 37	≥ 37
Birth weight, g	< 2500	≥ 2500	≥ 2500	≥ 2500	≥ 2500	≥ 2500	≥ 2500	≥ 2500
Death during sleep	+	+	+	+	+	+	+	+
Co-sleeping	–	–	–	+	+	+	–	+
Sleeping position	prone	supine	prone	lateral	prone	supine	prone	supine
Maternal age, years	20–34	35–	20–34	20–34	20–34	20–34	20–34	20–34
Number of siblings	1	0	0	≥ 2	1	1	0	≥ 2
Siblings with SUID	–	–	–	–	–	–	–	–
Breastfeeding	+	+	+	+	–	–	–	+
Passive smoking	+	–	–	–	+	–	–	+
Alcohol influence^b^	–	–	–	–	–	–	–	–
Vaccination within 1 month	+	+	–	–	–	–	–	–
Maltreatment	–	–	–	+	–	–	–	+
Infectious disease findings								
Clinical symptoms	–	–	–	–	–	+	–	–

^a^Diagnosis estimated with the highest probability matching the experts' diagnosis.

^b^Alcohol influence during childcare with caregivers. SIDS, sudden infant death syndrome; SUID, sudden unexpected infant death.

### A SIDS onset-predictive support model for effective prevention during different developmental phases

**Figure 4:**
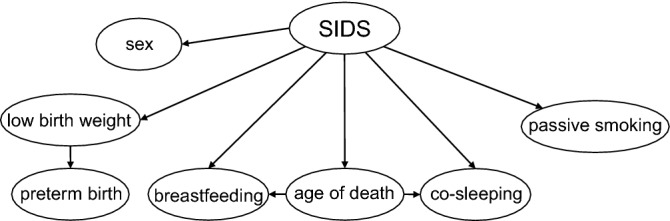
A Bayesian onset-predictive support model for SIDS. A conditional probability table was incorporated for each factor. Including the presence or absence of each factor produces a SIDS-onset probability as an annual incidence rate per 1000 of the population. The prior probability of annual SIDS incidence in the general population was 0.3/1000 live births calculated from vital statistics of a population survey^9,18^ and our 64 cases. SIDS, sudden infant death syndrome.

As a preliminary step to construct an SIDS onset-predictive support model, we first compared the demographic characteristics of 41 SIDS cases with the data of a healthy infant population (Table 5). In contrast to the results of the analysis of the SIDS diagnostic models, seven of nine major factors, with the exception of sex and gestational age, showed statistically significant changes for SIDS incidence. In the co-sleeping and breastfeeding groups, the risk of SIDS incidence differed considerably among the age groups (Table 5). Next, we constructed an onset-predictive support model using Bayesian approach, as described in the Methods section (Fig. 4). Table 6 shows the onset-prediction results estimated by inputting the presence or absence of passive smoking/co-sleeping according to the age group.

**Table 5:** Comparisons of demographic characteristics between SIDS and general infants.

	SIDS (n = 41)	Control^a^	*P* value^b^
	n (%)	n (%)	
**Age distribution**			
0–2 months	13 (31.7)	− (25.0)^c^	–
3–6 months	24 (58.5)	− (33.3)^c^	
7–11 months	4 (9.8)	− (41.7)^c^	
**Sex**			
Male	22 (53.7)	6,865,626 (51.3)	0.88
Female	19 (46.3)	6,518,337 (48.7)	
**Gestational age**			
< 37 weeks	4 (9.8)	762,547 (5.7)	0.29
≥ 37 weeks	37 (90.2)	12,617,333 (94.3)	
Unknown	0 (0.0)	4083 (0.0)	
**Birth weight**			
< 2500 g	10 (24.4)	1,276,948 (9.5)	0.004
≥ 2500 g	31 (75.6)	12,104,667 (90.5)	
Unknown	0 (0.0)	2348 (0.0)	
**Co-sleeping**			
*0–2 months*			
Yes	12 (91.7)	43 (30.4)	< 0.001
No	1 (8.3)	98 (69.6)	
*3–6 months*			
Yes	17 (70.8)	74 (53.5)	0.12
No	7 (29.2)	65 (46.5)	
*7–11 months*			
Yes	3 (75.0)	85 (60.6)	0.63
No	1 (25.0)	73 (39.4)	
**Sleeping position**			
Prone	19 (46.3)	422 (18.9)	< 0.001
Not prone	22 (53.7)	1816 (81.1)	
**Breastfeeding**			
*0–2 months*			
Yes	11 (84.6)	3555 (96.0)	0.09
No	2 (15.4)	148 (4.0)	
*3–6 months*			
Yes	7 (29.2)	4011 (85.1)	< 0.001
No	17 (70.8)	700 (14.9)	
*7–11 months*			
Yes	3 (75.0)	− (81.1)^d^	–
No	1 (25.0)	− (18.9)^d^	
**Passive smoking**			
Yes	21 (51.2)	− (33.1)^d^	–
No	9 (22.0)	− (66.9)^d^	
Unknown	11 (26.8)	–	

^a^Control of age, sex, gestational age, and birth weight were from the Japanese national vital statics database between 2006 to 2018^18^; co-sleeping was from Ichikawa et al.^29^; sleeping position was from Togari et al.^30^; breastfeeding was from the Japanese national nutrition survey on preschool children in 2015^31^; passive smoking was from the Prevalence of tobacco consumption by Japan Tobacco Incorporated in 2018^32^.

^b^Fisher’s exact test/Fisher-Freeman-Halton test, as appropriate.

^c^Each age group of the general infant population was considered as evenly distributed.

^d^Only the proportions are published. SIDS, sudden infant death syndrome.

**Table 6:** Comparison of annual SIDS incidence rates per 1000 live births estimated with a Bayesian onset-predictive support model, with and without passive smoking or co-sleeping by age.

Groups	A	B	C	D	Group D/Group A	Group D/general SIDS incidence^a^
Passive smoking	–	–	+	+		
Co-sleeping	–	+	–	+		
Age						
0–2 months	0.02	0.5	0.09	2.4	120.0	8.0
3–6 months	0.14	0.3	0.7	1.5	11.0	5.0
7–11 months	0.02	0.04	0.09	0.2	10.0	0.7

^a^The incidence was calculated from the total SUID incidence rate (0.49/1000 births/year) in Japan and 64% of SIDS proportion in 64 SUID cases in this study, which is 0.3/1000 births. SIDS, sudden infant death syndrome; SUID, sudden unexpected infant death.

The predicted incidence in all age groups increased with the addition of any risk factor. The risk of SIDS in 0- to 2-month-old infants with both risk factors was eightfold higher than that in the general infant population. Comparison of SIDS incidence with and without both risk factors revealed that the risk of SIDS was reduced by 120-fold when both risk factors were absent in 0- to 2-month-old infants. The models also revealed that the risk weight of passive smoking was lower than that of co-sleeping in the 0–2 month group (0.09 and 0.5, respectively). The opposite results were observed in the older age groups (0.7 and 0.3 in the 3–6 month group; 0.09 and 0.04 in the 7–11 month group, respectively), although the weight ratios were smaller than those in the 0–2 month group. The risk of SIDS was lower in the 7–11 month group than in the general infant population.

Supplementary Table S1 presents the results of the same calculation performed for non-breastfed male infants with low birth weight, which is a representative high-risk population. The probability of SIDS incidence was higher in all 12 subgroups compared with the results presented in Table 6 (approximately tenfold higher in the 0–2 month group and 3–6 month group and three-fold higher in the 7–11 month group). Almost identical values were noted for changes in SIDS incidence with and without passive smoking and co-sleeping in non-breastfed male infants with low birth weight (10.0 and 124.5, respectively) (Supplementary Table S1).

## Discussion

This is the first proof-of-concept study that demonstrated that Bayesian approach could be used to construct SIDS diagnostic and onset-predictive support models using a small number of SUID forensic autopsy cases with detailed information as training data (Figs. 3, 4)^17^. Our SIDS diagnostic support models reproduced the same diagnosis for most cases diagnosed by experts as SIDS (Table 4). The incidence estimated by our onset-predictive model increased with each addition of risk factors and was comparable to those previously reported by other research groups (Table 6 and Supplementary Table S1)^12,22^. Studies with larger sample sizes are generally more reliable than those with smaller sample sizes. However, in SIDS research, it is difficult to perform a study with a large sample size especially because of diagnostic inconsistencies^4,6,7^. Indeed, we observed that the classification of SUID cases in Japan was highly variable among regions based on general population data (Fig. 1).

To reduce the inconsistency in SIDS diagnosis, we used forensic autopsy cases diagnosed in a single institution as the study sample, and consensus diagnoses were made by a multidisciplinary team consisting of experts in the sudden-pediatric-death area. Forensic autopsy cases also have the advantage of providing detailed and accurate information from police death-scene investigations. Based on consensus diagnoses, SIDS accounted for 64% of SUID cases, which was consistent with the classification ratio reported in other high-income countries^4,9^.

In the validation analyses of our SIDS diagnostic support models, they indicated SIDS as the most likely diagnosis in four or five of six SIDS cases (Table 4). These results suggest that Bayesian approach may facilitate the development of diagnostic support models, even with a small number of forensic autopsy cases, using only data that can be obtained before autopsy.

Moreover, the probability values in cases with a diagnosis of SIDS identical to the diagnosis by experts ranged from 0.71 to 0.87 in Model 3, suggesting that the models also represent the uncertainty of the diagnosis of SIDS by experts. If a novel diagnostic method is developed in the near future, the model's diagnostic accuracy will improve in accordance with an improvement in the experts’ diagnostic accuracy. Therefore, if non-experts use this Bayesian-approach model, it will resolve the challenge of diagnostic variability. We believe that this would ultimately lead to the prevention of SIDS.

Nevertheless, non-matched diagnoses in some cases were noted, and no trauma cases were included in the present validation study. Moreover, despite using the Bayesian models constructed in this study, it is difficult for researchers to draw inferences about any differences in the background characteristics between “SIDS” and “Others” in Table 3. The Bayesian approach can be “updated” using additional data even after the model has been established. Thus, further research is warranted to improve the quality of SIDS diagnostic support models based on the Bayesian approach.

We also demonstrated that Bayesian approach was useful for developing onset-predictive support models for SIDS. The corresponding changes in the predicted incidence rate according to changes in risk indicated that this model could perform a proper risk assessment. The results in each subgroup with certain risk factors were comparable with those reported previously by other research groups, suggesting that the prediction probabilities estimated by Bayesian models in this study were realistic^12,22–24^. Models such as those constructed in this study could inform caregivers on measures to reduce the risk of SIDS.

In addition, the onset-predictive model demonstrated that each risk factor carried a different weight for SIDS development according to infant age. Co-sleeping had a higher risk weight than passive smoking among infants aged 0–2 months, whereas this pattern was reversed after 3 months of age (Table 6). These results suggest that the mechanism underlying SIDS may differ among different infant age groups. A recent study analyzing the Centers for Disease Control and Prevention Birth Cohort Linked Birth/Infant Death Data Set (2003–2013: 41,125,233 births and 37,624 SUIDs) reported that the risk of SUID associated with maternal smoking increased sharply after the first 48 h of birth, peaked on day 21, and plateaued at an approximately 1.4-fold risk over the first 6 months^12^. Another recent study used a similar data set and reported that maternal smoking during pregnancy doubled the risk of SUID^22^. Logistic regression models were used in both these studies, and the increased risk associated with smoking was similar to that reported in the present study. This also indicates that the Bayesian models constructed using a small number of cases with detailed information could reproduce prediction probabilities that were previously reported by other research groups^12,22–24^. Meanwhile, the modulatory effects of other factors on smoking-associated risk were not assessed in previous studies. In contrast, Bayesian approach enabled us to analyze changes in smoking-associated risk with the addition of other risk factors such as co-sleeping. Accordingly, Bayesian models may catalyze the discovery of novel mechanisms underscoring SIDS.

This study demonstrated that the diagnostic inconsistencies among regions in Japan (Fig. 1) were similar to those in other countries^4,7,9,10^. It is concerning that these inconsistencies may adversely impact the proposal of workable preventive measures by the Child Death Review (CDR), which commenced in 2020 as a pilot project in Japan. A recent report from the United Kingdom, which has a long history of CDR, revealed that the terms “SIDS” and “accidental asphyxia” are underused in the CDR, even in typical cases, and that there is wide variation in the subcategorization of SUID among professions^6^. In this regard, employing statistically objective numerical values of SIDS or non-SIDS probabilities using a diagnostic support model may improve decision-making processes for the CDR. We believe that the development of SIDS diagnostic support models may contribute to the unification of SUID diagnostic criteria and support the appropriate classification of causes of death by the CDR team, ultimately leading to the establishment of effective approaches for preventing future child deaths.

Despite the important clinical implications of our data, this study has several limitations. One of the major limitations was that we used a small number of autopsy cases from a single institution to construct the SIDS diagnostic and onset-predictive support models. The networks estimated too many parameters with only 64 observations. Therefore, it will be necessary to confirm whether SIDS diagnostic and onset-predictive support models can reproduce similar results using different cases at multiple other institutions. Another major limitation was that in constructing onset-predictive support models, we did not compare the background characteristics and risk factors associated with SIDS to those in surviving age-matched infants as controls from the same population the deaths occur. In contrast to the conventional approach, researchers could use multiple control groups in a single study considering the high flexibility of Bayesian inference. Therefore, to overcome the limitations due to the small number of patients in the SIDS groups, we used different control groups for multiple comparisons between the SIDS and general population groups (Fig. 4, Table 6). Nevertheless, future studies are warranted to confirm whether the models can show similar results using ideal controls.

In conclusion, to establish standardized diagnostic tools and effective preventive strategies for SIDS, we constructed SIDS diagnostic and onset-predictive support models based on the Bayesian approach using a small number of forensic autopsy cases at a single institution. Furthermore, the model found age-related differences in the risk of SIDS. We also identified considerable interregional heterogeneity in the SUID classification in Japan, which was associated with a high ratio of undetermined causes of death and a low ratio of SIDS diagnoses. Because of the complexity of risk factors and a small number of cases, SIDS is inherently difficult to diagnose and challenging to predict the onset. The current proof-of-concept study demonstrates that Bayesian approaches could be a promising tool for the establishment of novel diagnostic and predictive strategies for SIDS due to its flexibility and applicability in small sample size studies.

## Methods

### Descriptive and estimated analysis

#### SUID classification analysis according to Japanese prefectures

To investigate heterogeneity in SUID classifications among prefectures in Japan, the number of deaths and mortality rate during 2012–2018 according to the cause of death in those under 1 year of age in 47 prefectures were examined using vital statistics from population survey reports released by the Ministry of Health, Labour and Welfare^18^. The definition of SUID included the following cause-of-death categories: SIDS, accidental asphyxia, and unknown causes of death. SIDS corresponded to R95 (SIDS) in ICD-10; accidental asphyxia corresponded to the combination of W75 (accidental suffocation and strangulation in bed), W78 (inhalation of gastric contents), and W79 (inhalation and ingestion of food, causing respiratory tract obstruction); unknown causes of death corresponded to the combination of R96 (other sudden deaths, cause unknown), R98 (unattended deaths), and R99 (other ill-defined and unspecified causes of mortality) ^9,12,13^.

#### Autopsy case analysis

Cases under 1 year of age at the time of death were extracted from forensic autopsy cases at Kyoto University from January 2006 to December 2018. Exclusion criteria were cases with insufficient data owing to dissipation of information, stillborn cases, in-hospital deaths owing to congenital anomalies and perinatal conditions, deaths owing to traffic injuries, and cases for which postmortem examinations were performed > 1 week later (Fig. 2). SIDS was defined as cases corresponding to Category IA and IB according to the San Diego definition of SIDS, including those in infants younger than 1 year of age^3^. The diagnosis of internal or external causes of death was based on the circumstances of death, gross anatomical findings, histopathology, and additional investigations as needed.

Diagnoses were initially made by a certified pathologist and pediatrician (HH), a certified pathologist (HK), and two experimental pathologists (MM and CK). Among the cases, 31 (48.4%) were reviewed at case conferences comprising participants from multiple disciplines related to pediatric medicine, including a pediatric expert (TO) and radiologic expert (AY). Consensus review-based diagnoses were used as the final diagnoses. Clinical course, police investigation information, and autopsy findings were investigated for a detailed evaluation and diagnosis of each case. Clinical course and police investigation information included details of the circumstances at the time of death, parenting environment, socioeconomic risk factors, and autopsy findings, including gross, histological, toxicological, biochemical, bacteriological, and virological examinations.

The following SIDS-related risk factors were examined: age of death, sex, gestational age, birth weight, death during sleep, co-sleeping and posture in the case of death during sleep, mother’s age, number of cohabiting siblings, family history of SUID, breastfeeding, passive smoking, caregiver alcohol influence, vaccination history, presence of maltreatment, and clinical signs of infection (Table 3)^25,26^.

Co-sleeping was defined as the child and caregiver or other cohabitants sleeping on the same plane with no partition. Death during sleep was defined according to agreement by the caregiver and police. Siblings of any age included half-siblings. SUID family history was defined as having a probability of SUID based on caregiver reports, police information, and past autopsy records of the institution and was limited to siblings or half-siblings of the child. Breastfeeding was defined as either total or mixed feeding. Passive smoking was defined as smoking by at least one parent and/or a cohabiting adult. The effect of alcohol on the caregiver was defined based on caregiver reports or police information indicating that the caregiver cared for the child under the influence of alcohol. Vaccine history was defined as any vaccination within 1 month of death^27^. Maltreatment was defined as the presence of a clear or suspected history of abuse and/or neglect reported by police and other welfare agencies. Clinical signs of infection were defined as the presence of at least one of the following symptoms: fever, cough, runny nose, vomiting, and diarrhea within 1 week of death.

Dissections were performed as previously reported^28^. Histological inflammatory findings were considered as the presence of inflammatory cell infiltration in at least one of the following organs^28^: brain, meninges, salivary glands, thyroid, trachea, lungs, heart, liver, spleen, kidneys, adrenal glands, and gastrointestinal tract. Rapid diagnosis of infectious diseases was performed using a rapid diagnostic kit for 10 types of bacterial and viral antigens (hepatitis B virus, hepatitis C virus antibody, pneumococcus, mycoplasma, influenza A and B viruses, respiratory syncytial virus, adenovirus, rotavirus, and norovirus) using samples collected during the autopsy.

Age, sex, gestational age, birth weight, co-sleeping by age, sleeping position at discovery, breastfeeding by age, and passive smoking were compared between SIDS cases and infants in the general population (Table 5). Control data on age distribution, sex, gestational age, and birth weight were obtained from the Japanese national vital statistics database during 2006–2018^18^; co-sleeping data were obtained from the study by Ichikawa et al.^29^; sleeping position data were obtained from the study by Togari et al.^30^; breastfeeding data were obtained from the Japanese National Nutrition Survey on Preschool Children in 2015^31^; and passive smoking data were based on the prevalence of tobacco consumption obtained from Japan Tobacco Incorporated in 2018^32^.

#### Categories of variables

Variables were categorized as follows: age, 0–2 months, 3–6 months, and 7–11 months; gestational age, < 37 weeks or more; birth weight, < 2500 g or more; sleeping position, supine, prone, other positions, and unknown; maternal age, < 19 years, 20–34 years, and ≥ 35 years; and number of siblings, 0, 1, and 2 or more. Other items were categorized as present (positive) or absent (negative).

### Bayesian model construction

#### Construction of SIDS diagnostic support models

Bayesian networks are graphical models describing the conditional dependencies of variables with accompanying joint probabilities^33,34^. In Bayesian network calculations, a pre-generated conditional probability table (CPT) is applied to the corresponding node^35^. Our CPTs represented the conditional probability of each risk in SIDS and non-SIDS cases.

When constructing the diagnostic support model, we considered two possible directions for the relationship between factors and causes of death. One approach estimated causes of death from factors and reflected the causal relationship of the factors leading to death; the other approach estimated death-related factors from causes of death and reflected the retrospective estimation of prenatal factors that affected mortality (e.g., autopsy cases). We constructed one causal model (Model 1) and two retrospective estimation models (Models 2 and 3). The causes of death in Models 1 and 2 consisted of SIDS and non-SIDS, and those in Model 3 consisted of SIDS and other internal or external causes of death. All models were tested against the aforementioned consensus diagnoses. CPTs were constructed based on the consensus diagnoses and risk factors of 64 cases. BayoLink (NTT DATA Mathematical Systems Inc., Tokyo, Japan) was utilized, which automatically calculates conditional probabilities in a complex Bayesian network model. The position of each risk factor, as well as the number and direction of the arrows, were manually created in accordance with SUID pathophysiology^1,25,26^. We adopted a construction in which the probability of final diagnosis indicated the highest value when it matched the consensus diagnoses of experts as the final appropriate Bayesian network.

After model construction, we verified model reproducibility using eight new cases under 1 year of age as a verification cohort. Cases underwent forensic autopsies in our department during 2019–2020.

#### Construction of SIDS onset-predictive support models

To confirm the usefulness of the Bayesian approach in constructing onset-predictive support models, a Bayesian network was generated by extracting seven known risk factors for SIDS: age of death, sex, gestational age, birth weight, co-sleeping, breastfeeding, and passive smoking status (Fig. 4). In model construction, we prioritized the presence or absence of co-sleeping habits and smoking in caregivers, which pose the highest modifiable risk factors for SUID in Japan^9,36^. CPTs for each risk factor were prepared using data from SIDS cases at our institution and the general infant population obtained from the literature. The risk of developing SIDS was calculated by varying the presence or absence of co-sleeping and passive smoking for all age distributions. The other five variables were set to the neutral state to fluctuate with and without co-sleeping and second-hand exposure to smoke. The same calculation was performed for non-breastfed male infants with low birth weight, who constitute a representative high-risk population.

### Statistical analysis

Data are reported as n (%) or arithmetic mean ± standard deviation unless otherwise noted. All data were analyzed in R (The R Foundation, Vienna, Austria)” software^37,38^. *P* values of the 16 factors in Table 3 and nine factors in Table 5 were calculated using Fisher's exact test or Fisher-Freeman-Halton test, when appropriate. A *P*-value < 0.05 was considered statistically significant.

### Ethical statement

This study was approved by the Kyoto University Ethical Committee (registration number: No. 2935) and this approval is valid until March 31, 2023. This study was also conducted according to the ethical guidelines for clinical research according to the Declaration of Helsinki guidelines. We used the opt-out method for enrolment. Case records and information were anonymized before analysis such that individuals were unidentifiable. As all autopsies performed in this study were commissioned by the police and they prohibited forensic pathologists from contacting caregivers to avoid interference with the investigation, the requirement for written informed consent was waived by the Kyoto University Ethics Committee.

## Supplementary Information

Supplementary Information.

## Data Availability

The datasets generated and/or analyzed during the current study are available from the corresponding author on reasonable request.
