# Supplementary material for: A proof-of-concept study to construct Bayesian network decision models for supporting the categorization of sudden unexpected infant death
Source: Sci Rep. 2022 Jun 13;12:9773. doi: 10.1038/s41598-022-14044-w (PMC9192651; doi:10.1038/s41598-022-14044-w)
Supplement: Supplementary file 1 — Supplementary Information. [file 41598_2022_14044_MOESM1_ESM.pdf]

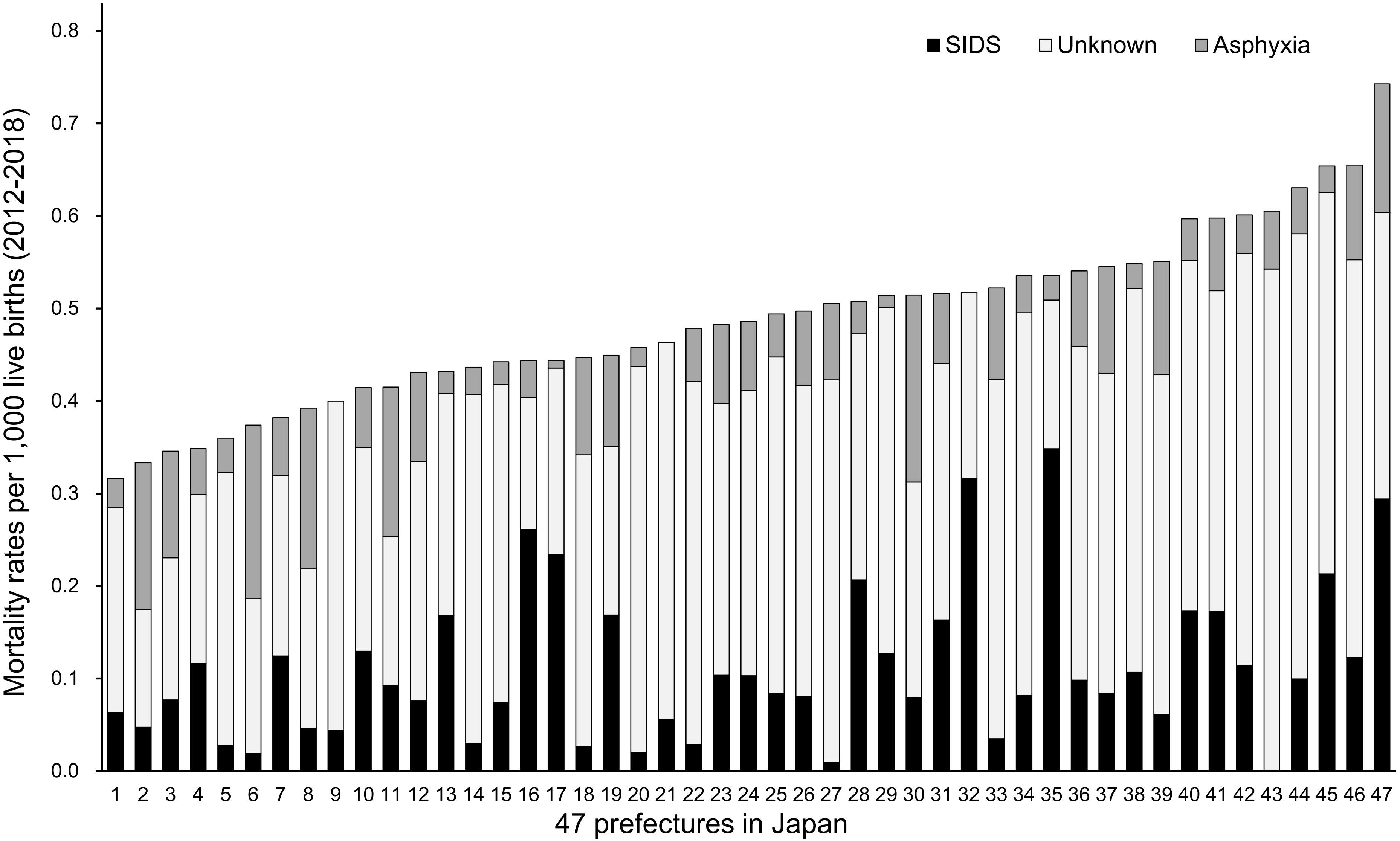

Supplementary Figure S1. Mortality rates due to sudden unexpected infant death (SUID) cases in the 47 prefectures in Japan from 2012 to 2018. SUID was defined as a set of the following three subcategories: SIDS, accidental asphyxia, and unknown causes of death. SIDS, R95 (SIDS) in ICD10; accidental asphyxia, the combination of W75 (accidental suffocation and strangulation in bed), W78 (inhalation of gastric contents), and W79 (inhalation and ingestion of food, causing obstruction of respiratory tract); unknown causes of death, the combination of R96 (other sudden death, cause unknown), R98 (unattended death), and R99 (other ill-defined and unspecified causes of mortality). ICD-10, International Classification of Diseases,10th Revision; SIDS, sudden infant death syndrome.

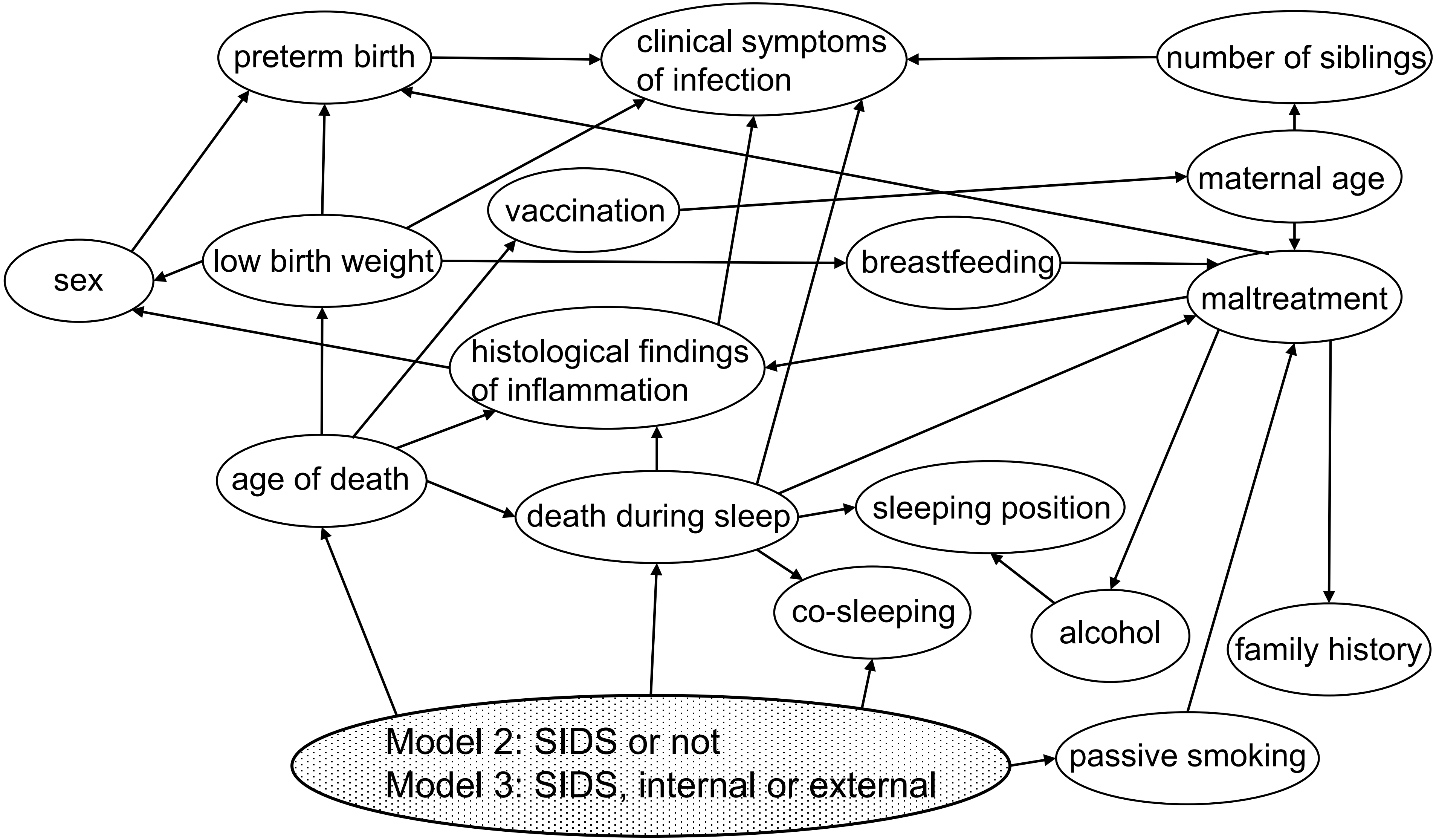

Supplementary Figure S2. Bayesian models for SIDS diagnosis support. This model reflects the retrospective estimation of the prenatal factors that affected the mortality (e.g., autopsy cases). A conditional probability table has already been incorporated for each factor. Entering the presence or absence of each factor will calculate the SIDS or other-cause of death diagnosis probability. SIDS, sudden infant death syndrome.

Supplementary Table S1. Comparison of annual SIDS incidence rates per 1,000 live births estimated with a Bayesian onset-predictive support model, with and without passive smoking or co-sleeping by age in case of low birth weight, non-breastfeeding male infants

Groups	A	B	C	D		
Passive smoking	–	–	+	+	Group D	Group D
Co-sleeping	–	+	–	+	/Group A	/general SIDS incidence[a]
Age						
0–2 months	0.2	5.4	0.9	24.9	124.5	83.0
3–6 months	1.9	4.0	8.9	18.7	9.8	62.3
7–11 months	0.07	0.14	0.3	0.7	10.0	2.3

[a]The incidence was calculated from the total SUID incidence rate (0.49/1,000 births/year) in Japan and 64% of SIDS proportion in 64 SUID cases in this study, which is 0.3/1,000 births. SIDS, sudden infant death syndrome; SUID, sudden unexpected infant death.
